# Shoulder pain, health-related quality of life and physical function in community-dwelling older adults

**DOI:** 10.3389/fragi.2023.1176706

**Published:** 2023-07-06

**Authors:** Derik L. Davis, Ranyah Almardawi, Brock A. Beamer, Alice S. Ryan, Michael L. Terrin

**Affiliations:** ^1^ Department of Diagnostic Radiology and Nuclear Medicine, University of Maryland School of Medicine, Baltimore, MD, United States; ^2^ Department of Epidemiology and Public Health, University of Maryland School of Medicine, Baltimore, MD, United States; ^3^ Department of Pathology, University of Maryland School of Medicine, Baltimore, MD, United States; ^4^ Department of Medicine, University of Maryland School of Medicine, Baltimore, MD, United States; ^5^ Baltimore Geriatric Research and Education Clinical Center (GRECC), Veterans Affairs Medical Center, Baltimore, MD, United States

**Keywords:** assessment, function, health-related quality of life, pain, shoulder

## Abstract

The impact of shoulder pain on health-related quality of life and physical function among community-dwelling older adults (>60 years) not seeking medical care is not well understood. Forty-four community-dwelling older adult volunteers with low comorbidity were stratified into two groups by the presence (*n* = 18) or absence (*n* = 26) of shoulder pain. Participants completed the 36-Item Short Form and American Shoulder and Elbow Surgeon surveys and received shoulder range of motion and magnetic resonance imaging testing. Participants with shoulder pain perceived more difficulty accomplishing usual tasks secondary to their physical and emotion health and displayed inferior shoulder function, relative to participants without shoulder pain. This study suggests that shoulder pain reduces quality of life and physical function in the population of community-dwelling older adults not seeking medical evaluation for their symptoms.

## Introduction

The shoulder is a common source of pain, affecting one in five adults in older populations (Patel et al., 2013). Pain is a significant burden to public health and diminishes older adults’ ability to maintain functional independence (Covinsky et al., 2009; Patel et al., 2013). The prevalence of shoulder pain is similar across age groups stratified by decade after age 65 years, and older women are slightly more likely to complain of shoulder pain relative to older men (Patel et al., 2013).

Shoulder pain is most frequently associated with rotator cuff tears involving the supraspinatus tendon, but not all painful shoulders are attributable to the rotator cuff (Chard et al., 1991; van der Windt et al., 1995; Melis et al., 2009; Tuite and Small, 2017). The specific impact of shoulder pain on quality of life and physical function in older populations not seeking care for their symptoms is not well understood (Bryant et al., 2007; Covinsky et al., 2009; Burner et al., 2014). Most studies describe shoulder pain’s effect on quality of life and physical function among clinical patients who pro-actively present for treatment of symptomatic shoulders (Vidt et al., 2016; Kukkonen et al., 2021; Meng et al., 2022). Currently, standard of care does not include routine health maintenance screening to identify undiagnosed shoulder disorders in older populations not actively self-reporting clinical symptoms. Thus, nearly one-half of older adults do not inform healthcare providers about their shoulder pain symptoms (Chard et al., 1991; Burner et al., 2014; Davis et al., 2022b).

The purpose of this brief research report is to provide a preliminary estimate of differences between community-dwelling older adult volunteers with shoulder pain who were not actively seeking treatment as compared to those without shoulder pain for metrics of health-related quality of life and shoulder physical function. We hypothesize that community-dwelling older adults with shoulder pain have diminished health-related quality of life and shoulder function as compared to older adults without shoulder pain.

## Methods

### Study design and participant characteristics

This study was approved by the University of Maryland Baltimore Institutional Review Board and complied with the Health Insurance Portability and Accountability Act. All participants provided written informed consent. This study is a cross-sectional analysis of a convenience sample of community-dwelling older adult volunteers who were recruited via advertising and enrolled into two separate pilot studies between 2017 and 2019 at the University of Maryland School of Medicine. Inclusion criteria for this study included an age between 60 and 85 years; enrollment in the control cohort of either pilot study; completion of shoulder MRI and shoulder range of motion testing; and completion of the American Shoulder and Elbow Surgeons (ASES) and SF-36 surveys. Men and women were included together in the study, since shoulder pain is similarly prevalent in each gender (Patel et al., 2013). Exclusion criteria for this study were history of rotator cuff repair surgery or joint replacement, contraindication to magnetic resonance imaging (MRI), and chronic upper extremity paralysis. The first pilot study was a longitudinal cohort study designed to compare supraspinatus intramuscular fatty infiltration and physical function between community-dwelling adult control volunteers and a population of patients with symptomatic full-thickness supraspinatus tendon tear of the rotator cuff eligible for care by orthopaedic surgeons (Davis et al., 2019). Data were included from 18 participants in the control cohort of the first pilot study’s second visit, when 36-Item short form (SF-36) surveys were first completed. The second pilot study was a longitudinal cohort study designed to compare supraspinatus intramuscular fatty infiltration and physical function between a different set of community-dwelling older adult control volunteers and symptomatic patients receiving physical therapy for initial management of presumptive rotator cuff tear (Davis et al., 2022a). Data were included from 26 participants in the control cohort of the second pilot study’s baseline visit when the SF-36 survey was first completed. Thus, this cross-sectional analysis includes data from a total of 44 participants.

### Measures

Shoulder MRI was performed at 3.0 T ipsilateral to the dominant hand in participants without shoulder pain; or, alternatively ipsilateral to the shoulder with pain. A board-certified musculoskeletal radiologist (DLD) independently classified each participant’s rotator cuff supraspinatus tendon into two categories: intact (normal or tendinopathy) or tear (partial-thickness tear or full-thickness tear) (Morag et al., 2006). On the same day as the MRI, participants completed all self-reported questionnaires and surveys, and shoulder physical examination testing. A medical history questionnaire included questions pertinent to record demographics, to list chronic conditions, and to calculate the Charlson co-morbidity index, inclusive of adding one point for every decade above the age 50 years (Charlson et al., 1987). Participants completed the SF-36 survey to estimate health-related quality of life and the ASES survey to determine self-report physical function of the shoulder ipsilateral to the MRI. The SF-36 survey has been validated to estimate self-reported perceptions of health-related quality of life and consists of eight subscales with each scale ranging from 100 (best quality of life) to 0 (worst quality of life) (Goldberg et al., 2001; McClure and Michener, 2003; Smith et al., 2012; Vidt et al., 2016). The ASES score is calculated based on a 1-item pain scale and 10 activities of daily living questions which are specific to shoulder function, such as level of difficulty reaching a high shelf, putting on a coat, or difficulty sleeping on the affected side. ASES score ranges from 100 (best shoulder function) to 0 (worst shoulder function). The ASES survey has been validated to quantify self-reported shoulder dysfunction in patients with a variety of shoulder disorders (McClure & Michener, 2003; Smith et al., 2012; Vidt et al., 2016). The pain score subcomponent in the ASES survey is based on an 11-point ordinal visual analog scale (VAS) for pain, ranging from 0 (minimum) to 10 (maximum). In the analysis, participants with a VAS ≤1 were classified as without shoulder pain, and VAS ≥2 were classified as having shoulder pain. (McMahon et al., 2014; Davis et al., 2019; Davis et al., 2021). Investigators have suggested that an ASES score ≥90 may be considered as the normal range of performance in the population of older adults without shoulder symptoms (Sallay and Reed, 2003; Moosmayer et al., 2009). A single examiner (RA) administered all self-reported surveys and performed shoulder forward flexion ROM and shoulder abduction ROM testing for all participants using a portable hand-held goniometer for the shoulder ipsilateral to the MRI (Davis et al., 2021; Davis et al., 2022a; Davis et al., 2022b). Height and weight were collected to calculate body mass index (BMI) in kg/m^2^.

### Statistical analysis

Descriptive statistics were performed as appropriate with continuous variables reported as mean ± standard deviation and categorical variables as counts and percentages. The descriptive characteristics between groups were compared with the unpaired *t*-test, Fisher’s exact test, and the chi-square test as appropriate. The heterogeneity of effect for age on the association between shoulder-pain status and outcomes were performed as appropriate. A *p*-value <0.05 was considered to indicate significance. Statistical analysis was performed using SAS statistical software version 9.4 (SAS, Cary, North Carolina).

## Results

The characteristics of the study population are listed in Table 1. The study population was well represented by both men (n = 21) and women (n = 23) who on average were overweight by BMI and showed relatively few comorbidities, with the mean Charlson co-morbidity index predominantly a function of age.

**TABLE 1:** Characteristics of the study population.

	Study population (n = 44)	- Shoulder pain (n = 26)	+ Shoulder Pain (n = 18)	*p*-Value
**Age, years**	69.3 ± 5.6^A^	67.4 ± 4.3	72.0 ± 6.3	**0.010**
**Male, %**	21 (47.7%)	15 (57.7%)	6 (33.3%)	0.112
**Body Mass Index, kg/m** ^**2**^	27.0 ± 5.5	27.9 ± 5.8	25.9 ± 4.9	0.298
**Charlson Co-Morbidity Index**	2.9 ± 1.0	2.9 ± 1.1	2.9 ± 0.9	0.934
**Co-morbidity**				
**Cancer**	0	0	0	1.000
**Cardiovascular Disease**	1 (2.3%)	1 (3.8%)	0	1.000
**Cerebrovascular Disease**	0	0	0	1.000
**Chronic Kidney Disease**	1 (2.3%)	1 (3.8%)	0	1.000
**Chronic Lung Disease**	1 (2.3%)	0	1 (5.6%)	0.409
**Connective Tissue Disease**	0	0	0	1.000
**Dementia**	0	0	0	1.000
**Diabetes Mellitus**	8 (18.2%)	6 (23.1%)	2 (11.1%)	0.439
**Liver Disease**	2 (4.5%)	1 (3.8%)	1 (5.6%)	1.000
**Peptic Ulcer Disease**	0	0	0	1.000
**Peripheral Vascular Disease**	1 (2.3%)	1 (3.8%)	0	1.000
**Supraspinatus tendon tear**	26 (59.1%)	13 (50.0%)	13 (72.2%)	0.141

Note. A = Values are mean ± standard deviation or n (%).—Shoulder Pain = shoulder pain is absent. + Shoulder Pain = shoulder pain is present. *p*-Values in bold are statistically significant (*p* < 0.05).

Participants with shoulder pain were older (*p* = 0.010). There were no significant differences between groups for mean percent male, BMI, co-morbidity, or frequency of supraspinatus tendon tear on MRI. Significant differences were present between groups for mean ASES score (*p* < 0.001), mean shoulder forward flexion ROM (*p* = 0.003), and mean shoulder abduction ROM (*p* = 0.001) in that those with shoulder pain had lower ASES score and reduced shoulder ROM (Table 2).

**TABLE 2:** Comparison of shoulder range of motion, self-reported shoulder function and health-related quality of life between participants with and without shoulder pain.

	Study population (n = 44)	- Shoulder pain (n = 26)	+ Shoulder Pain (n = 18)	*p*-Value
**Shoulder range of motion**				
**Forward Flexion,°**	150.5 ± 27.0^A^	160.9 ± 15.1	135.8 ± 33.5	**0.003**
**Abduction,°**	141.0 ± 28.6	152.7 ± 18.3	124.2 ± 32.7	**0.001**
**ASES** ^**B**^ **Score**	86.5 ± 16.1	96.5 ± 5.1	72.0 ± 15.6	**< 0.001**
**SF-36 Subscales**				
**Physical Functioning**	83.2 ± 19.9	86.7 ± 19.3	78.1 ± 20.2	0.195
**Limits to Physical Health** ^**C**^	79.5 ± 36.3	90.4 ± 27.5	63.9 ± 42.2	**0.037**
**Limits to Emotional Health** ^**D**^	89.3 ± 27.6	98.5 ± 6.6	75.9 ± 39.3	**0.028**
**Vitality**	69.8 ± 17.5	73.3 ± 17.2	64.7 ± 17.1	0.191
**Emotional Wellbeing**	84.6 ± 13.7	86.0 ± 13.7	82.7 ± 14.0	0.133
**Social Functioning**	92.3 ± 15.5	95.2 ± 13.3	88.2 ± 17.9	0.194
**Pain**	75.7 ± 20.9	80.6 ± 19.4	68.8 ± 21.6	0.106
**General Health**	78.2 ± 18.0	81.7 ± 17.9	73.1 ± 17.4	0.129
**Health Change**	56.3 ± 21.0	53.9 ± 18.3	59.7 ± 24.5	0.243

Note. A = Values are mean ± standard deviation or n (%). B = American Shoulder Elbow Surgeons. C = Role Limitations Due to Physical Health. D = Role Limitations Due to Emotional Health.—Shoulder Pain = shoulder pain is absent. + Shoulder Pain = shoulder pain is present. *p*-Values in bold are statistically significant (*p* < 0.05).

For the subscales of the SF-36 survey, significant differences in mean scores were present between groups for role limitations due to physical health (*p* = 0.037) and role limitations due to emotional health (*p* = 0.028). No significant differences were found for the other SF-36 subscales.

Since participants with shoulder pain were older relative to those without pain, additional analyses were performed to determine the heterogeneity of the age effect on outcomes. Age had no significant interaction with shoulder-pain status (presence or absence of shoulder pain) for the outcomes of ASES score (*p* = 0.723), role limitations due to physical health score (*p* = 0.560), role limitations due to emotional health score (*p* = 0.425), forward flexion ROM (*p* = 0.536) or abduction ROM (*p* = 0.954).

## Discussion

This brief research report suggests that community-dwelling older adults with shoulder pain have significantly inferior health-related quality of life related to role limitations due to physical health and role limitations due to emotional health subscales in the SF-36 survey, as compared to those without shoulder pain. This study also found that community-dwelling older adults with shoulder pain demonstrate significantly inferior shoulder physical function by the self-reported ASES score and shoulder forward flexion ROM and abduction ROM on physical examination. The novelty of this study is that these study findings pertain to a study population of community-dwelling older adult volunteers with relatively few comorbidities who were not seeking medical care for their shoulders.

Nearly 41% of our volunteer older adult participants reported shoulder pain, in line with prior studies suggesting that shoulder pain is a burden for older populations. There was a nearly 20% prevalence of shoulder pain in the 2011 National Health and Aging Trends Study, a study of 7601 participants designed to estimate the burden of pain among the broader population of older adults in the United States (Patel et al., 2013). The higher percentage of older women reporting shoulder pain in our study sample, relative to older men, is also consistent with findings of the NHATS (Patel et al., 2013). Even active, high-functioning older adults not seeking medical care experience shoulder pain. McMahon et al., 2014 reported that 24% of 141 older adult athletes participating in the 2005 Senior Olympics complained of shoulder pain on self-reported questionnaires. The reason for our study’s higher prevalence of shoulder pain is unclear, although selection bias was possible due to self-referral of participants, in response to local advertisement, who may have been eager to join the study for evaluation at no cost. Older adult volunteer participants with shoulder pain in our sample were older than those without pain, differing from NHATS which reported that participants aged 70–74-year had a 1.5% lower prevalence of shoulder pain relative to those aged 65–69 years. There were no significant differences between groups in our sample for BMI, and older adult volunteer participants with and without shoulder pain were on average overweight (U.S. Centers for Disease Control and Prevention, 2022).

Perception of one’s ability to accomplish tasks influences self-reported quality of life. Participants without shoulder pain in this study demonstrated a ceiling effect for the SF-36 subscales of role limitations due to physical health and role limitations due to emotional health, signaling the absence of perceived barrier(s) in completing usual tasks. By contrast, participants with shoulder pain reported significantly inferior scores and greater variability, reflecting perceptions of difficulty for performing usual tasks. Our results align with others who report that musculoskeletal pain contributes to poorer quality of life (Uchida et al., 2020). As expected, participants with shoulder pain also demonstrated lower ASES scores and inferior shoulder ROM in agreement with Covinsky et al., 2009 who found that older adults with pain have greater risk for upper extremity functional limitations.

This study is not without limitations. Although the sample size was small and sample sizes were not equal between those with and without shoulder pain, some statistically significant differences for characteristics between groups were identified. The study population was a convenience sample, which may be limited by selection bias and results may not be generalizable to the general population. Participants with shoulder pain were older compared to participants without shoulder pain, although age had no significant interaction with shoulder-pain status for outcomes. The study design did not examine differences in physical activity levels between groups. The study did not evaluate duration of pain or differences in pain at rest *versus* with physical activity for the shoulder pain group or assess the contralateral shoulder by MRI. As this study occurred at one time point, future studies are needed to evaluate the impact of changes in shoulder pain on physical function and health-related quality of life over time in older populations. The study population also demonstrated an overall low burden of comorbidity, and the impact of shoulder pain may be different in older populations with a higher burden of comorbidity.

In conclusion, this study suggests that shoulder pain may contribute to a decrease in health-related quality of life and reduced physical function in older adult populations not seeking medical care for shoulder pain. Larger future studies are warranted to determine the longitudinal effect of shoulder pain on health-related quality of life and physical function for the population of community-dwelling older adults not seeking medical care for their shoulder pain.

## Data Availability

The original contributions presented in the study are included in the article/supplementary material, further inquiries can be directed to the corresponding author/s.
